# Protocol for quantifying the adhesive strength of single fluorescent cells adhering to a non-fluorescent cell monolayer by fluidic force microscopy

**DOI:** 10.1016/j.mex.2026.103855

**Published:** 2026-03-06

**Authors:** Gubesh Gunaratnam, Eugene Oh, Philipp Jung, Markus Bischoff, Wolfgang Metzger

**Affiliations:** a Institute of Medical Microbiology and Hygiene, Saarland University, Building 43 66421, Homburg, Germany; b Department of Trauma, Hand and Reconstructive Surgery, Saarland University, Building 57 66421, Homburg, Germany

**Keywords:** Fluid force microscopy, Biomechanics, Cell-cell-interaction, Cell Adhesion

## Abstract

Fluidic force microscopy combines the strengths of microfluidics and atomic force microscopy, allowing the analysis of cell detachment with up to several hundred nanonewton adhesion forces, without the application of chemical adhesives, which is not possible with atomic force microscopy alone. Fluidic force microscopy has been used in a variety of cell detachment studies where the cell of interest is attached to the substrate or embedded in cell monolayers. However, it might be difficult to study cells in vertical overlays with this method because they belong to adjacent vertical layers and cannot be easily identified with brightfield microscopy. Therefore, we present here a detailed protocol for the detection of adherent, fluorescent single cells on a non-fluorescent cell monolayer and for testing detachment levels using the fluidic force microscopy setup. This protocol allows the quantification of high nanonewton adhesion forces and has the potential to be applied to other types of adherent cell lines.

## Highlights

• Fluidic force microscopy allows the analysis of cell detachment without chemical adhesives.
• A study of detachment of vertically overlaid cells might be difficult due to limited visibility of brightfield microscopy.
• This protocol presents an easy to implement method to quantify adhesion forces between cells.

## Graphical abstract

**Figure:**
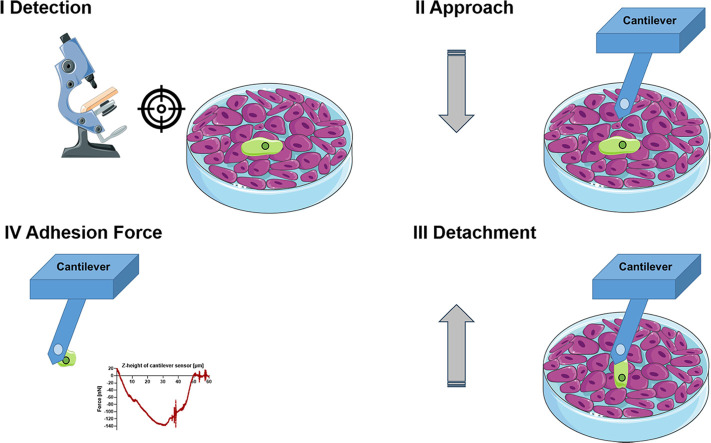

## Specifications table

**Table:** 

**Subject area**	Biochemistry, Genetics and Molecular Biology
**More specific subject area**	Biomechanics, Cell-Cell-Interaction, Cell Adhesion
**Name of your protocol**	Protocol for quantifying the adhesive strength of single fluorescent cellsadhering to a non-fluorescent cell monolayer by fluidic force microscopy
**Reagents/tools**	1. Fluid FM Platform For FluidFM-based SCFS, we used an AFM system consisting of a Nanosurf FlexAFM (Nanosurf AG, Liestal, Switzerland) and a Cytosurge FluidFM module (Cytosurge AG, Glattbrugg, Switzerland). The system is commercially available, but the FluidFM module is also compatible with Nanosurf DriveAFM or various AFMs from Bruker (e.g. JPK Nanowizard V, Bruker, Berlin, Germany). 2. FluidFM micropipette The following components are part of our FluidFM system: 1. FlexAFM V5 scan head 2. Nanosurf C3000 controller 3. Nanosurf I100 scan head interface 4. LC400 DSP piezo controller 5. Sample stage for inverted microscopes 6. Joystick for controller x and y motors (all Nanosurf AG, Liestal, Switzerland) 7. Cytosurge Microfluidic control system V2 (Cytosurge AG, Glattbrugg, Switzerland) 8. Halcyonics.i4 - active vibration isolation (Park Systems GmbH, Göttingen, Germany) 9. Zeiss Axio Observer Z1 10. Zeiss AxioCam 11. Light source HXP 120 V and different filter sets (Zeiss filter set 00 (for propidium iodide detection), 38 HE (for GFP detection) and 49 (for DAPI detection)) (all Zeiss AG, Oberkochen, Germany) 3. FluidFM micropipette The FluidFM micropipette (Cytosurge AG) is the essential cantilever construct for detachment of single adherent eukaryotic cells from the cell monolayer. Various micropipette apertures and stiffnesses (spring constants) are available. The aperture connects the cantilever microchannel to the external liquid environment and is critical for suction activity to apply negative pressure to the cell. We used a micropipette with an 8 µm aperture, which covers a large area of the cell during the contact phase. Available spring constants for micropipettes with this aperture are 0.3 N/m, 2 N/m and 4 N/m. In preliminary experiments, we found that the 0.3 N/m spring constant was too soft to resolve the adhesion forces in our cell-cell interactions, so we switched to 2 N/m. Using a micropipette with the latter spring constant, we were able to completely resolve the adhesion forces. The micropipette reservoir was filled with 10 µl of a glycerol solution, which slowed down the clogging process inside the micropipette. To mount the FluidFM micropipette on the AFM system and use it, the following components were required: 1. MAT FluidFM Micropipette (Aperture: 8 µm, Spring constant: 2 N/m) 2. FluidFM Pneumatic Connector 3. FluidFM Cantilever Holder for measurements in liquid (all Cytosurge AG, Glattbrugg, Switzerland) 4. Filtered 50 % glycerol in autoclaved and salt-free water. 5. Autoclaved and filtered salt-free water 4. Cell culture Although fibroblasts were used in this FluidFM study, any adherently growing cell or cell line can be studied using this protocol. For the FluidFM experiments, cell monolayers of the selected cells are seeded in a thin glass bottom dish with good optical resolution. Another batch of cells is stained with a fluorescent label before being overlaid on the cell monolayer. For this protocol, we have chosen a commercially available donor cell line, cryopreserved Normal Human Dermal Fibroblasts (NHDF). The following equipment is required: 1. NHDF (lot 488Z008.2, PromoCell GmbH, Heidelberg, Germany) 2. Fluorodish FD 5040 glass dish (World Precision Instruments, Sarasota, FL; U.S.A.) 3. CellTracker™ Green CMFDA (Thermo Fisher Scientific, Eugene, OR, USA) 4. Cell culture flask (Growth area 7500 mm2, Greiner Bio-One, Frickenhausen, Germany) 5. Dulbecco's modified Eagle's medium (DMEM, Sigma-Aldrich, Taufkirchen, Germany 6. HEPES (Carl Roth GmbH + Co. KG, Karlsruhe, Germany) 7. Fetal calf serum (FCS, PanBiotech, Aidenbach, Germany) 8. 6-well cell-culture plate (Greiner Bio-One, Frickenhausen, Germany) 9. Sterile-filtered FluidFM medium (DMEM+15 % FCS+25 mM HEPES) 5. Software Different programs are needed to control the AFM operations and the optical microscope, and to process and quantify the AFM data. 1. Nanosurf C 3000 version 3.10.5.14 (Nanosurf AG, Liestal, Switzerland) 2. Zeiss Zen 2 lite (Zeiss AG, Oberkochen, Germany) 3. Image Metrology SPIP version 6.6.5 (Digital Surf, Besançon, Frankreich) 4. OriginLab OriginPro version 2019b (Northampton, MA, USA)
**Experimental design**	Single fluorescent cells were seeded on a non-fluorescent cell monolayer. A Fluid FM apparatus was used to quantify the adherent strength between the single, fluorescent cell and the cell monolayer.
**Trial registration**	None
**Ethics**	Not applicable, only commercially available cells were used
Value of the Protocol	- This protocol allows the measurement of adhesion strength between cells in an efficient way - The fluorescent marker CMFDA allows the detection of single cells which adhere on a cell monolayer - The Fluid FM apparatus is able to quantify the adhesion strength between two adherent cells

## Background

It is widely accepted that spheroids are better suited to mimic the physiological conditions of cells in tissues than traditional 2D cultures where cells adhere to artificial surfaces [1]. Biomechanically, spheroids act as a link between cells and tissues [2]. To model spheroid biomechanics mathematically, quantifying cell-cell adhesion strength is essential. A simple method is to create a confluent 2D monolayer and overlay it with single cells of the same type for a defined adhesion time. To our knowledge, a detailed protocol for measuring these interactions has not yet been described.

Fluidic force microscopy (FluidFM)-based single cell force spectroscopy (SCFS) has been widely used to study the adhesion forces of single eukaryotic cells on artificial substrates [[3], [4], [5]] or within cell monolayers [6,7]. Both scenarios are based on different modes of adhesion. While only the underlying cell’s binding capacity is influenced by the substrate, the substrate is not part of direct cell-cell interaction. These interactions depend on the glycocalyx or adhesive transmembrane proteins [8]. Overlying cells also encounter intercellular extracellular matrix (ECM) and ECM fiber orientation strongly influences cell orientation and adhesion strength [9].

The FluidFM combines atomic force microscopy (AFM) principles with a microfluidic control system (MFCS) [10] and has been used in numerous cell adhesion studies [[11], [12], [13], [14], [15], [16], [17]]. A recently published review provides a detailed overview of FluidFM and a thorough description of its fundamental principles and biological applications [18].The core component is the FluidFM cantilever, which integrates a microchannel connecting the MFCS to the external liquid environment through an aperture. MFCS controls pressure like a pipette: positive pressure pushes, negative pressure pulls. The cantilever, acting as a micropipette, applies negative pressure to grab a cell and attach it. Retraction detaches the cell from the sample surface, allowing adhesion force quantification..

Studying cell-cell interactions require single-cell detection and strong suction up to 3.5 µN (−700 mbar at an 8 µm aperture) [3]. Depending on cell type and experimental conditions, individual cells on top of a cell layer may be identifiable in brightfield microscopy without fluorescence labelling [19]. If the seeded cells differ from those forming the layer, morphological differences can allow clear discrimination. However, cells adhering only briefly may not yet show final morphology. Fluorescent labelling of cells seeded on a layer additionally enables verification that the cell of interest was successfully detached.

Commercial AFM or FluidFM systems can integrate fluorescence microscopes and filter sets. The fastest way to detect fluorescent cells is via eyepiecesequipped with a dual-axis scale to align the micropipette with the fluorescent cell. Once centered, the micropipette is positioned vertically over the cell. Detachment can be observed live in brightfield mode, revealing cell body and protrusion separation, aiding interpretation of force-distance curves. After detachment, lateral micropipette movement checks completeness. Switching back to fluorescence confirms the cell is attached to the micropipette and correctly removed.

This paper provides a protocol for FluidFM users to detect fluorescent single cells on a monolayer and quantify adhesion strength using a commercial FluidFM setup on an inverted fluorescence microscope.

## Description of protocol

**Fig. 1:**
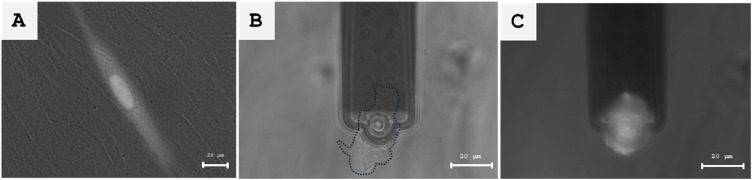
(A) Cell detection: CMFDA-stained fluorescent single cell on a non-fluorescent cell monolayer as seen with eyepieces. (B) FluidFM-based SCFS: Fluorescent cell detached from the cell monolayer by SCFS observed in brightfield mode. The closed, dashed line indicates the contour of the cell. (C) Detachment control: Detached cell immobilized on the micropipette and seen in fluorescent mode. For demonstration purposes pictures have been taken with the camera and with a 63x oil objective. Scale bar 20 µm.
  6. Set a force setpoint of 20 nN to 50 nN and click the "Approach" button in the AFM software to allow the final, force-controlled approach of the micropipette to the cell monolayer. Note: The setpoint should be as low as possible. If the set point range is not sufficient to reach the surface, this might indicate a low laser signal or errors during calibration (see 4.13).
  7. Switch from the camera to the eyepieces. Select the 50x magnification.
  8. Set the stage height to −20 µm to allow free movement of the micropipette.
  9. Align the opening of the micropipette with the cross line of the dual-axis measuring scale of one of the eyepieces using the manual X/Y axis adjustments of the stage.
  10. Press and hold the "Retract" button for 1–2 s to retract the micropipette. Do not retract too far or the micropipette will be lifted out of the liquid in the dish.
  11. Select the fluorescence mode and the appropriate filter (e.g. Zeiss 38 HE for CMFDA detection).
  12. Search for a fluorescent cell by moving the AFM stage with the joystick.
  13. "Approach" the micropipette next to the fluorescent cell with a force set to 20 nN.
  14. Reduce the stage height position from −20 µm to −100 µm.
  15. Bring the cross line of the eyepiece, and thus the opening of the micropipette, to a central position above the cell.
  16. Click "Approach" to bring the micropipette into contact with the fluorescent cell.
  17. Quickly check in brightfield mode if the micropipette is still centered on the crossline. If not, center the micropipette slightly and click "Approach" again.
  18. Switch to camera and brightfield mode.
  19. Start force-distance measurements with following parameters:
    a. Ramp height: 100 µm
    b. Approach and retract speed: 2 µm/s (Range: 1 µm/s – 10 µm/s)
    c. Ramp time: 50 s (Correlates with the ramp height and speed)
    d. Data points: 50,000
    e. Force value: 20 nN (10 nN – 70 nN)
    f. Contact time: 5 s
    g. Negative pressure and time: −700 mbar for 55 s (starting with contact)
  20. Observe the SCFS process and live view of cell detachment through the microscope camera.
  21. Check if any elongated cell protrusions from the cell are detached. This will aid in the interpretation of the force curve.
  22. After the cell is detached, carefully move the stage in a lateral direction using the joystick. Look for anchoring points of the cell with the cell monolayer (see Fig. 1B). If there are none, the cell is completely detached.
  23. Switch to the eyepieces and fluorescence mode.
  24. Slowly move the micropipette with the immobilized cell in a lateral direction. If it fluoresces, the previously selected cell has been detached and the experiment was successful.
  25. Save the force-distance curve (Fig. 2) for post-processing.

1. Cultivation of fibroblasts
  1. Thaw cryopreserved fibroblasts (500,000 cells/vial) and seed them into a T75cell culture flask in DMEM with 15 % FCS.
  2. Cultivate the cells at 37 °C, 95 % humidity, 5 % CO2 and change medium three times a week until the reach sub-confluence.
  3. To generate confluent cell monolayers, seed the fibroblasts at a seeding density of 110 cells/mm2 in a volume of 2.5 ml DMEM supplemented with 15 % FCS in a fluorodish (day −2).
  4. To prepare fluorescent single cells for overlay, seed 50 cells/mm2 of the same passage in a volume of 3 ml into a cavity of a 6 well cell culture plate (day −2). Cells are stained with a fluorophore the following day (day −1).
2. Fluorescence staining of cells
  1. On the day of the experiment (day 0), wash the cells in the 6 well cell culture plate (see 1.) with sterile PBS after removing the cell culture medium.
  2. Add pre-warmed fluorescence staining solution consisting of 20 µM CMFDA in serum-free DMEM for 1 h incubation time.
  3. Remove the staining solution and rinse the cell layer twice with PBS. Add DMEM with 15 % FCS for 1 h incubation time.
3. Seeding of CMFDA-labeled cells on a confluent cell monolayer
  1. Trypsinize the CMFDA-labeled cells and seed them at a seeding density of 4 cells/mm2 on the confluent cell layer in the fluorodish.
  2. Allow cells to adhere for 3 h in DMEM with 15 % FCS before FluidFM experiments.
4. Calibration of the FluidFM system
  1. Turn on the AFM controller and other AFM-related electronics. Turn on the microfluidic control system, the computer and the optical microscope.
  2. Start the AFM software and the optical microscope control software..
  3. Wait (usually up to an hour) to allow the AFM laser photodiode enough time to stabilize and reach optimum power.
  4. Place the micropipette on the cantilever holder. Fill the liquid reservoir at the back of the micropipette with 10 µl 50 % glycerol. Connect the FluidFM pneumatic connector to the micropipette. Mount the micropipette on the AFM scan head.
  5. Switch to the camera of the optical microscope and use the 10x magnification objective to bring the micropipette into the field of view. Make sure that the micropipette is mounted evenly on the AFM scan head.
  6. Reduce the light from the microscope (i.e., so that you can clearly see the laser appearing on the display). Place the laser on the open end of the micropipette to obtain maximum laser intensity.
  7. Tilt the photodiode of the AFM to bring the reflection of the laser into its center.
  8. Calibrate the spring constant of the micropipette in air using the thermal tune function built into the AFM software.
  9. Press the glycerol from the liquid reservoir into the inner microchannel of the micropipette at a pressure of 1 bar. Glycerol will usually exit the tip of the micropipette after 1.5 - 2 min, which is a good indication that the micropipette is functional. Remove the AFM scan head from the stage.
  10. Place a fresh fluorodish on the stage. Pipette 50 µl FluidFM medium (composition see Reagents/Tools) into the dish. Place the AFM scan head back on the stage. Make sure that the micropipette is now completely immersed in the fluid. If not, carefully pipette a small drop of medium between the micropipette and the glass of the cantilever holder to help cover the micropipette completely with liquid.
  11. Allow the micropipette to equilibrate in the liquid. Due to the different refractive indices of air and liquid, the laser must be repositioned on the micropipette. Then tilt the photodiode to bring the laser back to the center.
  12. Approach the micropipette to the surface of the fluorodish using the automatic control of the AFM software. Calibrate the crosstalk and deflection sensitivity.
  13. The micropipette is now calibrated for experiments. Do not move the laser position between calibration and experiments, as this can introduce errors into the force data. Tip: To reduce errors during spring constant and deflection sensitivity calibrations, refer to the work of Nagy et al. for guidance [20]. Additionally, Mulder et al. describe solutions to some problems that may occur during micropipette preparation in detail [21].
5. FluidFM-based SCFS
  1. Replace the cell supernatant in the dish with 1 ml of FluidFM medium. Reduce bleaching of stained cells by minimizing ambient light sources.
  2. Place and fix the dish on the AFM stage.
  3. Mount the AFM scan head with the attached micropipette over the dish. Verify that the micropipette is now fully embedded in the liquid of the dish by visual inspection and by checking the laser signal intensity in the photodiode window of the AFM software.
  4. Switch to camera mode and focus on the cells using the 10x magnification objective.
  5. Manually approach the micropipette and the glass surface of the dish using the "Advance" button of the AFM software. Stop before the micropipette is in focus. A blurred view is sufficient (see Fig. 1A).

**Fig. 2:**
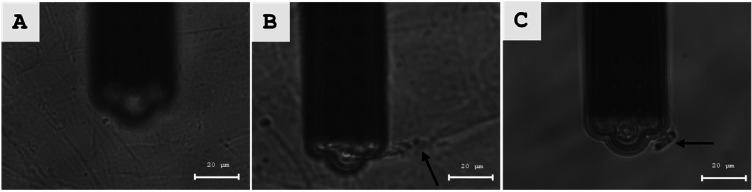
(A) Micropipette closed to the glass dish substrate. Manual approach was stopped when a blurry vision of the micropipette was reached. For precise, force-controlled contact between both surfaces, the automatic approach followed next. (B) Left: An individual cell kept a major anchoring point with the cell monolayer after SCFS completion (black arrow). Right: Manual retraction of the micropipette detached the remaining anchoring point of the cell from the cell monolayer (black arrow). For demonstration reasons pictures have been taken with the camera and with a 63x oil objective. Scale bar 20 µm.
6. Cleaning and storage of FluidFM probes
  The micropipette was treated with a cleaning solution and washed with water between each SCFS measurement. It was then stored in the refrigerator under dry conditions for reuse. The cleaning and storage procedures are summarized below:
  1. Prepare a small (approx. 50 µl) drop of cleaning solution (filtered 5 % sodium hypochlorite (Hedinger, Stuttgart, Germany)) in a fluorodish.
  2. Prepare a second fluorodish with three 50 µl drops of salt-free water, ensuring there is sufficient distance between them. Prepare a third fluorodish with a single 50 µl drop of FluidFM medium.
  3. Place the dish with the cleaning solution on the AFM stage to immerse the mounted micropipette. Apply a negative pressure of −50 mbar for 10 s and a positive pressure of 100 mbar for 10 s to clean the interior of the micropipette and then expel the cleaning solution.
  4. Remove the micropipette from the cleaning solution, carefully removing any visible excess solution from the cantilever holder with a Kimtech wipe (Kimberly-Clark Corporation, Irving, TX, USA), taking care not to touch the micropipette.
  5. Transfer the micropipette into the different liquids of water, one after the other, to remove the cleaning solution completely. Then, dry the micropipette again with a Kimtech wipe.
  6. Equilibrate the micropipette in FluidFM medium before using it again for SCFS. Alternatively, for long-term use (up to 4 weeks depending on usage frequency), store the dried micropipette in the original cantilever package of the manufacturer, seal the package with a sealing film, and transfer it into a sealed container in the refrigerator.
7. Post processing and adhesion parameters
  1. Open the raw data curve as a force-distance (e.g. height) curve in the processing software (e.g. SPIP).
  2. Perform baseline corrections on the approach curve. The baseline is important to define the "0 nN" base value and to quantify the adhesion forces. Tip: Perform a baseline analysis in a separate preliminary curve measured on glass to confirm a small difference between the approach and retract curve baselines (see Fig. 4A). Due to technical limitations, the retract curve may not be fully resolved in some cell-cell detachment experiments.
  3. Save the curve as an ASCII file.
  4. Open the ASCII file in Microsoft Excel and transform the height (X) and force (Y) values into your analysis software (e.g., OrginPro).
  5. Integrate the area limited by the retract curve and the baseline to obtain the adhesion energy and read the negative value for the adhesion force (see Fig. 3B). If the entire cell detachment process has been recorded, the last negative force value represents the cell detachment length on the x-axis.

**Fig. 3:**
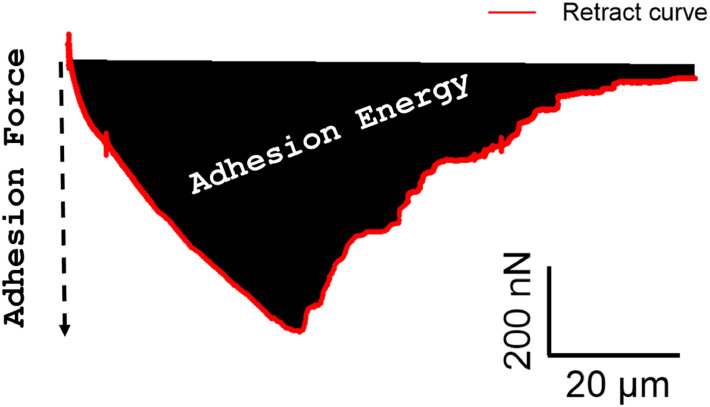
Exemplary retract force-distance curve and detachment events of a single fibroblast NHDF cell from a NHDF cell monolayer. The adhesion force is the minimum value and marks the strongest resistant of the cell to detachment. The adhesion energy is the integral of all detachment forces.

**Fig. 4:**
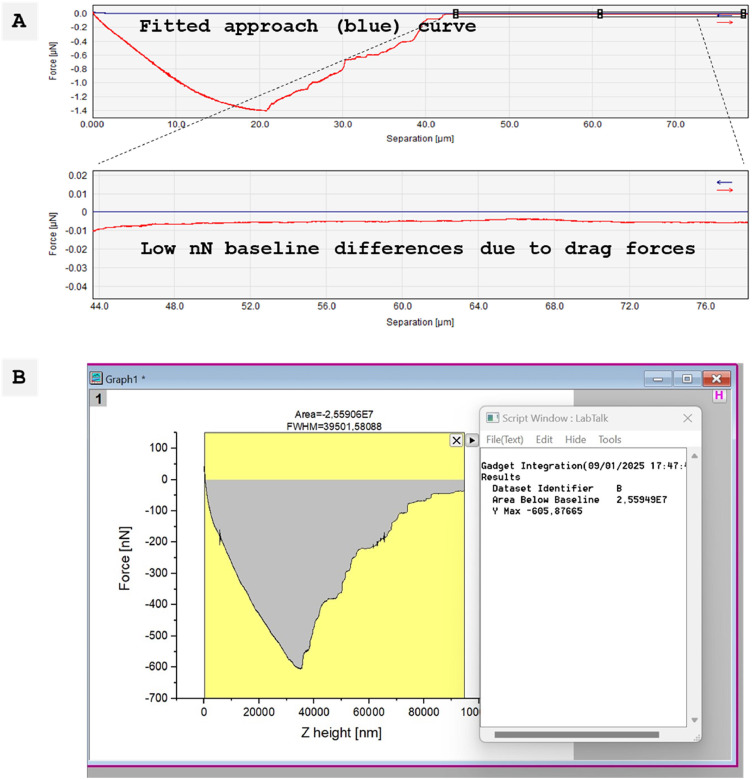
(A) Baseline analysis in a preliminary experiment of a single fluorescent fibroblast that was detached from a glass substrate. The approach force-distance curve was fitted to receive the “0 nN” base force level. Approach curve was compared to the retract curve for baseline deviation. (B) Adhesion analysis of a cell-cell interaction between a fluorescent fibroblast and a non-fluorescent cell monolayer based on approach-curve baseline fit.

## Protocol validation

None.

## Limitations

None.

## Supplementary material *and/or* additional information

No additional supplementary material and/or additional information.

## CRediT authorship contribution statement

**Gubesh Gunaratnam:** Conceptualization, Investigation, Methodology, Writing – original draft, Writing – review & editing. **Eugene Oh:** Conceptualization, Investigation, Methodology, Writing – original draft, Writing – review & editing. **Philipp Jung:** Funding acquisition, Writing – review & editing. **Markus Bischoff:** Funding acquisition, Writing – review & editing. **Wolfgang Metzger:** Conceptualization, Funding acquisition, Investigation, Methodology, Writing – original draft, Writing – review & editing.

## Declaration of competing interest

The authors declare that they have no known competing financial interests or personal relationships that could have appeared to influence the work reported in this paper.

## Data Availability

Data will be made available on request.
